# Themes and Variations: Regulation of RpoN-Dependent Flagellar Genes across Diverse Bacterial Species

**DOI:** 10.1155/2014/681754

**Published:** 2014-01-02

**Authors:** Jennifer Tsang, Timothy R. Hoover

**Affiliations:** Department of Microbiology, University of Georgia, Athens, GA 30602, USA

## Abstract

Flagellar biogenesis in bacteria is a complex process in which the transcription of dozens of structural and regulatory genes is coordinated with the assembly of the flagellum. Although the overall process of flagellar biogenesis is conserved among bacteria, the mechanisms used to regulate flagellar gene expression vary greatly among different bacterial species. Many bacteria use the alternative sigma factor **σ** ^54^ (also known as RpoN) to transcribe specific sets of flagellar genes. These bacteria include members of the Epsilonproteobacteria (e.g., *Helicobacter pylori* and *Campylobacter jejuni*), Gammaproteobacteria (e.g., *Vibrio* and *Pseudomonas* species), and Alphaproteobacteria (e.g., *Caulobacter crescentus*). This review characterizes the flagellar transcriptional hierarchies in these bacteria and examines what is known about how flagellar gene regulation is linked with other processes including growth phase, quorum sensing, and host colonization.

## 1. Introduction

The flagellum is an exquisitely complex nanomachine that is the primary means for motility in many bacteria. Given that motility plays a vital role in important microbial processes such as chemotaxis, host colonization, and biofilm formation, understanding how bacteria regulate flagellar biogenesis is critical for developing new strategies for the control of harmful microbes and manipulation of useful ones. Flagellar biogenesis is a highly ordered process that involves the coordinated regulation of dozens of structural and regulatory genes with the assembly of the flagellum. As expected for choreographing such an intricate process, flagellar biogenesis involves some of the most sophisticated regulatory mechanisms found in microbiology.

**Figure 1:**
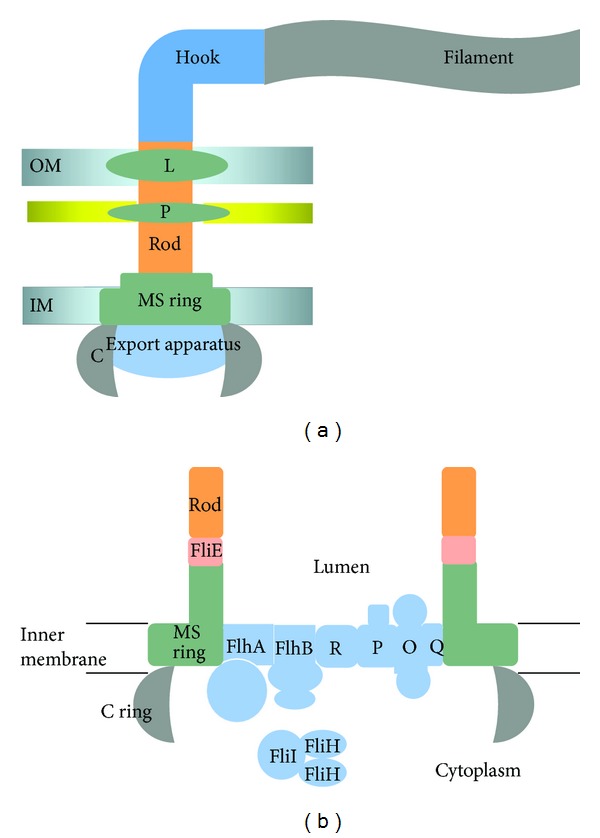
Structure of the flagellum. (a) An overview of the structure of the flagellum in Gram-negative bacteria. Abbreviations are as follows: L ring (L), P ring (P), outer membrane (OM), inner membrane (IM), and C ring (C). (b) Components of the flagellar basal body. Export apparatus proteins are shown in light blue. Abbreviations of the export apparatus proteins are as follows: FliR (R), FliP (P), FliO (O), and FliQ (Q). Details of the organization of the export apparatus proteins are not known, although results from genetic studies suggest associations between FlhA and the MS ring [10], FlhB and FliR [11], and FliO and FliP [12]. FliI is an ATPase which forms a heterotrimer with FliH. These proteins function together with other chaperones (not shown) to shuttle protein substrates to the export pore. Upon docking with a platform formed by the large cytoplasmic domains of FlhA and FlhB, a larger FliI_6_FliH_12_ complex is formed. In most bacteria the C ring is composed of three different types of protein subunits (FliG, FliM, and FliN). The C ring in *Salmonella* contains an estimated 26 copies of FliG, 34 copies of FliM, and ~136 copies of FliN. FliG is closest to the membrane and interacts with the MS ring, while FliN is the most distal to the membrane and FliM is situated between FliG and FliN. The *H. pylori *C ring contains FliG, FliM, and FliN plus an additional protein subunit (FliY) that shares homology with FliN [13]. Additional information on the bacterial flagellar protein export apparatus can be found in reviews by Minamino et al. [1] and Macnab [14].

Although the structure of the flagellum differs slightly between Gram-negative type and Gram-positive type bacteria, in all cases the bacterial flagellum is comprised of three main parts: the basal body, hook, and filament [1]. Components of the basal body are located within or are closely associated with the cell envelope. The basal body consists of several distinct structures, including the C ring, a type III secretion system known as the flagellar protein export apparatus, the flagellar motor, the rod, and rings that anchor the flagellum to the membrane (Figure 1(a)). Both Gram-negative and Gram-positive type bacteria possess MS and P rings, located in the inner membrane and peptidoglycan layer, respectively. Gram-negative type bacteria possess another ring known as the L ring located in the outer membrane. These rings provide support for the rod as it goes through the cell envelope. Depending on the bacterial species, the C ring consists of a complex of three or four different types of protein subunits assembled around the export pore of the flagellar protein export apparatus and extending into the cytoplasm. The C ring has a role in switching the rotational direction of the flagellum but has an additional role in directing protein substrates to the flagellar protein export apparatus for transport [2]. The flagellar protein export apparatus (Figure 1(b)) transports axial components of the flagellum (i.e., proteins that constitute the rod, hook, and filament) across the cell membrane where they are incorporated at the distal end of the nascent flagellum [1, 3, 4].

Despite the commonality in flagellar architecture, the ways in which bacteria regulate expression of their flagellar genes vary remarkably (reviewed by Smith and Hoover [5] and Anderson et al. [6]). This review focuses on systems which utilize the alternative sigma factor *σ* ^54^ (also known as RpoN) for regulating transcription of specific sets of flagellar genes. Sigma factors bind core RNA polymerase and allow the resulting RNA polymerase holoenzyme to recognize specific promoter sequences [7]. All bacteria possess a primary sigma factor (RpoD; referred to as *σ* ^70^ in *E. coli*) and most also utilize one or more alternative sigma factors for transcription of specific sets of genes. A unique feature of RpoN-dependent transcription is the requirement of an activator to stimulate the transition of a closed complex between *σ* ^54^-RNA polymerase holoenzyme (*σ* ^54^-holoenzyme) and the promoter to an open promoter complex that is able to initiate transcription [8, 9]. *Escherichia coli* and *Salmonella* are the archetypes for flagellar biogenesis and gene regulation, but these bacteria do not use RpoN for transcription of flagellar genes. Nevertheless, we refer to the *E. coli*/*Salmonella* systems throughout the review to make inferences for other systems that are not as well characterized. The bacteria that utilize RpoN in flagellar gene expression constitute a diverse group of microorganisms, some of which are human or animal pathogens. We discuss aspects of flagellar gene regulation in response to host and environmental stimuli and potential ramifications of these responses for survival and persistence of the bacteria.

## 2. Transcriptional Hierarchies Governing Flagellar Gene Expression

**Figure 2:**
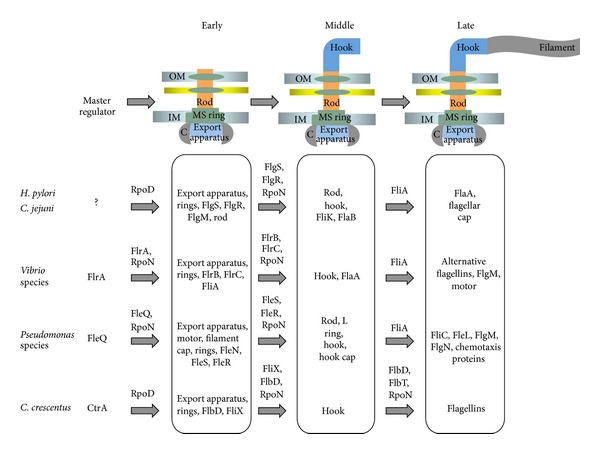
Diversity in flagellar gene transcription hierarchies. Transcriptional hierarchies are compared between the Epsilonproteobacteria, *Vibrio*, *Pseudomonas,* and *C. crescentus*. Regulatory proteins and sigma factors involved in controlling the transcriptional hierarchies are indicated above the arrows. Early, middle, and late genes are indicated between the arrows.

Though microorganisms utilize various mechanisms to control flagellar gene expression, in most (if not all) bacteria the flagellar genes are transcribed in an organized fashion where genes that encode components needed early in flagellar biogenesis are transcribed before genes encoding proteins required later in the assembly process (Figure 2). To a certain extent these transcriptional hierarchies are governed by the organization of the flagellar genes into different regulons based on the sigma factors required for their transcription. These transcriptional hierarchies are further coordinated by regulatory proteins that control expression of various sets or classes of genes. However, the mechanism by which such a hierarchy is regulated in a given bacterial species is not always understood and varies among organisms. A master regulator(s) initiates the flagellar gene transcription cascade and is generally thought to couple flagellar biogenesis to the cell cycle. For some bacteria, however, master regulators have yet to be identified. The master regulator stimulates transcription of the early genes which generally encode components of the basal body as well as an array of regulatory proteins which, depending on the bacterium, includes RpoN, FliA (*σ* ^28^), FlgM (an anti-*σ* ^28^ factor), or other proteins. Following completion of the basal body and hook, late genes encoding components of the filament are expressed.

### 2.1. *H. pylori* and *C. jejuni*

The best studied members of the Epsilonproteobacteria are *Campylobacter jejuni* and *Helicobacter pylori*. *C. jejuni* is a food-borne pathogen that colonizes the intestinal tract where it causes severe diarrhea, while *H. pylori* colonizes the stomach where it can cause peptic ulcer disease which can progress into gastric cancer if left untreated. Both organisms synthesize polar flagella that are required for colonization of the host [15–18]. Moreover, the degree of motility appears to be important for *H. pylori* virulence as more motile strains are able to maintain higher bacterial density and inflammation in the stomach cardia than those that are less motile [19].

In *H. pylori* and *C. jejuni*, flagellar gene regulation involves the primary sigma factor RpoD, as well as the alternative sigma factors RpoN and FliA (Figure 2). A master regulator which activates transcription of the early flagellar genes has not been identified for *H. pylori* or *C. jejuni*. It is possible that these bacteria lack a true master regulator of flagellar biogenesis, a possibility that was suggested by Niehus and coworkers since motility is required for the obligate parasitic lifestyle of *H. pylori* [20]. Alternatively, *H. pylori* or *C. jejuni* could possess a master regulator for flagellar biogenesis that has other essential roles which might explain why it has not been identified from mutagenesis screens.

The organization of flagellar genes into regulons based on the sigma factor required for transcription is very similar, though not identical, in *H. pylori* and *C. jejuni*. RpoD is required for transcription of genes encoding components of the basal body as well as regulatory proteins that control transcription of genes required at later stages of flagellar biogenesis [20]. Other genes regulated by RpoD include *flhF* and *flhG* whose products influence the localization of flagella to the cell pole and number of flagella per cell, respectively [21–23]. It is not known if transcription of the early flagellar genes is temporally regulated and intimately associated with the cell cycle as occurs in *E. coli *and *Salmonella* [24, 25] or if these genes are transcribed constitutively. Distinguishing between these two options would require one to follow temporal changes of early flagellar genes transcription in synchronous cultures, which has not been reported.

Transcription of genes needed midway through flagellar biogenesis is dependent on RpoN. These genes encode the proximal rod proteins, the hook protein, hook-associated proteins, the hook-length control protein (FliK), a minor flagellin (FlaB), and enzymes required for glycosylation of the flagellins [20, 26, 27]. The transcription of the RpoN-dependent genes is activated by a two-component regulatory system consisting of the sensor kinase FlgS and the response regulator FlgR [26, 28–30]. FlgS differs from most sensor kinases in that it is not membrane bound. The signal or cellular cue to which FlgS responds has yet to be identified, but several studies (see below) have implicated the flagellar protein export apparatus in this process. FlgS uses ATP to autophosphorylate a specific histidine residue in response to the cellular cue, and this phosphate is subsequently transferred to a specific aspartate residue in FlgR to activate it. Based on studies with other RpoN-dependent activators (reviewed by Bush and Dixon [31]), phosphorylation of FlgR likely stimulates oligomerization of the protein from a dimer to a hexamer capable of activating transcription. RpoN-dependent activators typically possess an N-terminal regulatory domain (often a response regulator domain as is the case in FlgR), an AAA+ ATPase domain that engages *σ* ^54^ and couples the hydrolysis of ATP with open complex formation, and a C-terminal DNA-binding domain that binds an enhancer sequence (reviewed by Schumacher et al. [32]). *H. pylori* FlgR is unusual in that it lacks a C-terminal DNA-binding domain and activates transcription by contacting *σ* ^54^-holoenzyme in the closed promoter complex without binding DNA [29]. Although *C. jejuni* FlgR possesses a C-terminal domain, it is not needed for activation of the RpoN regulon suggesting that, like its counterpart in *H. pylori*, *C. jejuni* FlgR engages the closed promoter complex from solution [33]. While the C-terminal domain of *C. jejuni *FlgR does not appear to play a role in DNA binding, it does prevent phosphorylation of the protein by acetyl phosphate which can interfere with normal flagellation [34].

The flagellar protein export apparatus has a major role in regulating expression of the RpoN regulon of *H. pylori* and *C. jejuni* as evidenced by the observation that mutations which interfere with formation of the export apparatus inhibit expression of RpoN-dependent flagellar genes. Porwollik and coworkers showed that inactivation of *fliI* or *fliQ* (encode a cytosolic ATPase component and integral membrane component of the export apparatus, resp.) in *H. pylori* results in reduced levels of the minor flagellin, FlaB, and the hook protein, FlgE, both of which depend on RpoN for their expression [35]. Allan and colleagues showed that mutations in *flhB* (encodes an integral membrane component of the export apparatus) result in reduced levels of FlaB and FlgE in *H. pylori* [36]; Smith and coworkers subsequently demonstrated that disrupting *flhB* inhibited expression of RpoN-dependent reporter genes [37]. Using DNA microarrays, Niehus and coworkers [20] demonstrated that FlhA (an integral membrane component of the export apparatus) is required for transcription of the entire RpoN regulon. Hendrixson et al. [38] reported that deleting any one of several integral membrane components of the export apparatus (FlhA, FlhB, FliP, or FliR) inhibited expression of an RpoN-dependent flagellar reporter gene in *C. jejuni*. Studies by Tsang and coworkers revealed that *H. pylori* strains which stably express truncated forms of FlhA are able to transcribe RpoN-dependent genes at levels that are close to wild type but are unable to export flagellar protein substrates [39]. Thus, any model for how the export apparatus influences transcription of the RpoN regulon must take into account that the export apparatus need not transport protein substrates to stimulate the RpoN regulon. Boll and Hendrixson recently showed that loss of the MS ring protein FliF or the C ring protein FliG inhibits transcription of RpoN-dependent flagellar genes in *C. jejuni *[40]. The MS ring is the first flagellar structure assembled and, in its absence, the export apparatus presumably is not formed which would account for the inhibition of the RpoN regulon in the *fliF* mutant. FliG may facilitate autophosphorylation of FlgS either by directly stimulating FlgS or by promoting assembly of the export apparatus into a conformation that stimulates FlgS activity [40].

The export apparatus is an ideal indicator for the status of flagellar assembly as it undergoes a major conformational change upon completion of the hook-basal body structure. In the early stages of flagellar biogenesis the export apparatus is in a conformation that transports rod- and hook-type substrates. Upon completion of the mature hook-basal body structure, the export apparatus undergoes a conformational change which is accompanied by a switch in substrate specificity to filament-type substrates [41]. This conformational change involves an autocleavage event in the C-terminal, cytoplasmic domain of the export apparatus protein FlhB (referred to as FlhB_C_), and interactions between the C-terminal domain of the hook-length control protein FliK and FlhB_C_ [42]. The change in conformation and substrate specificity of the export apparatus may serve as a cellular cue for the temporal regulation of flagellar genes. Indeed, in *E. coli* and *Salmonella* the anti-*σ* ^28^ factor FlgM is a filament-type substrate and is secreted by the export apparatus upon completion of the mature hook-basal body structure which allows transcription of the *σ* ^28^-dependent flagellar genes to occur [43].

Transcription of the RpoN-dependent flagellar genes in *H. pylori* also requires the putative RpoN chaperone FlgZ (HP0958) [44, 45]. FlgZ was first implicated in having a role in flagellar biogenesis in *H. pylori* when it was identified as interacting with RpoN in a high-throughput screen of a yeast two-hybrid system (https://pim.hybrigenics.com/) [46]. In the absence of FlgZ, RpoN is turned over rapidly in *H. pylori* with a half-life of about 30 minutes compared to a half-life greater than 4 hours in wild type [45]. The molecular basis for how FlgZ protects RpoN from rapid turnover is not known, but it is possible that FlgZ binds RpoN to protect it from proteolysis or that FlgZ facilitates the association of RpoN with core RNA polymerase which serves to protect RpoN from degradation. Overexpression of RpoN restores motility in a *H. pylori flgZ* mutant indicating that FlgZ is not essential for flagellar biogenesis [45]. In addition to its potential role as an RpoN chaperone, FlgZ may have additional roles in flagellar biogenesis as it was also shown to interact with the flagellar export apparatus protein FliH in the yeast two-hybrid system (https://pim.hybrigenics.com/) [46]. In addition, Douillard and coworkers demonstrated that FlgZ binds the *flaA* transcript and is needed for optimal expression of FlaA [47]. These researchers proposed that FlgZ functions with FliH to direct *flaA* transcripts to the flagellar protein export apparatus where translation of the transcripts could be coupled with secretion of the nascent FlaA [47].

Transcription of genes needed late in flagellar biogenesis in *H. pylori* and *C. jejuni*, which includes genes encoding the major flagellin, filament cap, and associated chaperones, is dependent on *σ* ^28^ (FliA) [20]. Similar to *E. coli* and *Salmonella*, the *H. pylori* FliA regulon is negatively regulated by FlgM [48]. Unlike *E. coli* and *Salmonella*, the inhibitory effect of FlgM on FliA in *H. pylori* is thought to be alleviated via interactions between FlgM and the C-terminal, cytoplasmic domain of FlhA (FlhA_C_) rather than by secretion of FlgM via the export apparatus [49]. *C. jejuni* also possesses a FlgM homolog [50], but unlike *H. pylori*, *C. jejuni* FlgM appears to be secreted from the cell via the flagellar export apparatus [51]. Interestingly, interaction of *C. jejuni* FlgM with FliA is temperature dependent, occurring at 42°C (the optimal growth temperature for *C. jejuni*) but not 37°C [51]. The primary function of *C. jejuni* FlgM is not to inhibit FliA activity during formation of the hook-basal body structure but rather to limit the length of the flagellar filament by suppressing expression of the FliA-dependent flagellin (FlaA) and the RpoN-dependent flagellin (FlaB) [51]. The mechanism by which FlgM represses transcription of RpoN-dependent genes in *C. jejuni* is unknown, but Wösten and coworkers have speculated that accumulation of the FliA/FlgM complex may inhibit phosphorylation of FlgS and/or FlgR [51]. FlgM also appears to inhibit transcription of RpoN-dependent genes in *H. pylori*, but so far this has only been demonstrated in an *flhA* mutant background [20].

### 2.2. *Vibrio* Species

The *vibrios* are typically saltwater microorganisms and several species cause food-borne illnesses. *Vibrio* species vary greatly in flagella morphology. *Vibrio cholerae *has a single polar sheathed flagellum, while *Vibrio parahaemolyticus* and *Vibrio alginolyticus* produce a single polar sheathed flagellum and lateral, peritrichous flagella, and *Vibrio fischeri* has a tuft of polar sheathed flagella. Lateral flagella promote swarming and colonization of surfaces and thereby enhance biofilm formation upon encountering highly viscous media or solid surfaces.

The flagellar gene transcriptional hierarchy in *Vibrio cholerae* is governed by RpoN and FliA [52]. *flrA* encodes an RpoN dependent activator and is the master regulator for the flagellar gene transcriptional hierarchy [52]. FlrA is regulated by cyclic di-GMP, a secondary messenger with roles in biofilm formation and virulence, which binds to FlrA and prevents it from binding the *flrBC* promoter [53]. Early and middle genes are RpoN-dependent but they rely on different transcriptional activators for their transcription. FlrA, along with RpoN, is required for transcription of the early genes which encode the components of the MS ring, C ring, and export apparatus. Other early genes encode the regulatory proteins FlrB, FlrC, and FliA. FlrB and FlrC constitute the sensor kinase and response regulator, respectively, of a two-component system that regulates transcription of the middle genes in conjunction with RpoN [54]. The middle genes encode the basal body-hook structures and the core flagellin, FlaA. The late genes are dependent on FliA for their transcription and encode alternative flagellins, FlgM, and components of the flagellar motor. As in *Salmonella*, FlgM is secreted via the flagellar protein export apparatus to relieve the inhibition on FliA activity in *V. cholerae* [55]. *flgA* is located upstream of *flgM* and its promoter (RpoN independent and FliA independent) may drive transcription of *flgM* such that FliA is repressed until formation of the mature hook-basal body structure [52].

The signal(s) that stimulates the FlrB/FlrC two-component system is not known. FlrD is necessary for transcription of middle and late flagellar genes, making it a candidate for regulating the FlrB/FlrC two-component system [56]. Additionally, FlrD contains HAMP domains (domain present in histidine kinases, adenyl cyclases, methyl-accepting proteins, and phosphatases) that are usually found in integral membrane proteins that are part of signal transduction pathways [56, 57]. Moisi and coworkers showed that FlrD is inserted into the inner membrane and proposed that it senses the completion of the MS ring-switch-export apparatus structure and communicates this information to FlrB [56].

The regulation of flagellar genes in *V*. *parahaemolyticus* is more complex since the bacterium is capable of producing lateral flagella in addition to polar flagella (reviewed by Merino et al. [58] and McCarter [59]). The polar flagellar system (Fla) is constitutively expressed while the lateral flagellar system (Laf) is expressed upon impedance of the polar flagella. The lateral flagella are used for swarming motility and enhance biofilm formation and host colonization by the bacterium [60, 61]. When grown planktonically, the bacterium produces a polar flagellum but when grown on highly viscous or solid medium [58] or during iron-limiting conditions [62], the bacterium produces lateral flagella. Thus, the polar flagellum acts as a mechanosensor to regulate expression of the lateral flagella genes through a mechanism that has yet to be defined. The dual flagellar system suited for locomotion under different conditions allows *V. parahaemolyticus* to be highly adaptive to changing habitats, including planktonic environments, surfaces, and biofilms.

The polar and lateral flagellar systems do not share any structural or regulatory components (aside from RpoN), but the regulatory networks that control polar and lateral flagellar systems in *V*. *parahaemolyticus* are similar to that of *V*. *cholerae*. The lateral flagellar genes are activated by LafK and, like the polar flagellar system, lateral flagellar genes are both RpoN dependent and FliA dependent [63]. Although LafK is homologous to master regulators of other flagellar systems, LafK does not appear to be a master regulator for the lateral flagellar system as the *fliM*_L_ operon (contains genes encoding components of the C ring and export apparatus) is not LafK dependent [64]. This suggests that there is another level of regulation preceding expression of these genes.

### 2.3. *Pseudomonas* Species

*Pseudomonas aeruginosa* is an aerobic Gram-negative Gammaproteobacterium that causes opportunistic infections, particularly in patients with cystic fibrosis where it results in inflammation and sepsis (reviewed by Veesenmeyer et al. [65] and Balasubramanian et al. [66]). *P*. *aeruginosa*, which thrives on surfaces and forms biofilms, is a prevalent agent of nosocomial infections. Wild-type *P*. *aeruginosa* cells form biofilms which can cause chronic infections. *P*. *aeruginosa* possesses a single polar flagellum that is required for virulence [67]. Motile strains of *P*. *aeruginosa* induce activation of the inflammasome (a multiprotein oligomer responsible for the activation of the inflammatory response) whereas nonmotile strains have a markedly reduced ability to induce inflammasome activation [68]. Nonmotile strains of *P*. *aeruginosa* may have an advantage during chronic infection by evading stimulation of the innate immune responses. However, a nonmotile *flgK* mutant is defective in surface attachment suggesting a role for flagella and/or motility in the initial cell-to-surface interaction [69]. Mutants in *fliM* and *cheY* are also nonmotile and are unable to form biofilm cap structures on initial biofilm colonies [70]. Other *Pseudomonas* species include *Pseudomonas fluorescens* which possesses multiple flagella and is found in the soil and water. Some *P*. *fluorescens *strains have been shown to protect plant roots against fungal infections [71]. *P.  fluorescens *does not typically cause disease in humans, but it is an opportunistic pathogen in immunocompromised individuals [72, 73].

Like the Epsilonproteobacteria and *Vibrio*, *P*. *aeruginosa* possesses a four-tiered transcriptional hierarchy for regulating flagellar gene expression. FleQ is the master regulator for flagellar synthesis and *fleQ* is regulated by global factors outside the flagellar regulons, such as cyclic di-GMP, a signaling molecule important in modulating the transition between planktonic and biofilm lifestyles [74]. The early flagellar genes are dependent on FleQ and RpoN for their transcription and encode components of the basal body as well as the filament cap, which is somewhat surprising since it is not needed until late in flagellar biogenesis [75]. The early genes also encode regulatory proteins which include *flhF*, *fleN*, *fleS,* and *fleR* [75, 76]. FleN is an antiactivator of FleQ that maintains the normal flagellum copy number of one per cell by downregulating genes encoding early flagellar components via a negative feedback mechanism [77, 78]. FleS and FleR form a two-component system that is required for the transcription of the middle RpoN-dependent genes [75]. The signal(s) required for FleS activation is unknown. The middle genes encode components of the basal body, including rod, L ring, hook, hook cap, and hook-filament junction proteins [75]. The late genes are FliA dependent and encode FliC (flagellin), FleL (filament length control protein), FlgM, FlgN (a protein required for initiation of filament assembly), and some chemotaxis proteins [75]. Transcription of *fliA* is constitutive [75], and FlgM (whose transcription is also FleQ dependent, but not RpoN dependent) represses the activity of FliA until completion of the hook-basal body structure, at which point it is secreted from the cytoplasm via the export apparatus which alleviates its inhibition of FliA.

### 2.4. *Caulobacter crescentus*

*Caulobacter crescentus* is an Alphaproteobacterium found in freshwater that is a model organism for cell cycle studies. *C*. *crescentus* divides asymmetrically to produce two cells with distinct morphologies: a swarmer cell and a stalked cell. The nonmotile stalked cell contains a polar stalk which secretes an exopolysaccharide that facilitates adhesion to surfaces. In addition, the stalked cell initiates DNA replication upon the start of cell division. In contrast, the swarmer cell is motile via a polar flagellum and DNA replication is repressed for a defined length of time until the cell differentiates into a stalked cell. Flagellar biogenesis is coordinated with the cell division cycle such that all progeny swarmer cells possess a functional flagellum.

Flagellar genes in *C*. *crescentus* are regulated by two sigma factors. The initiation of DNA replication in the swarmer cell results in expression and activation of the global transcription factor CtrA. CtrA also acts as the master regulator for flagellar gene expression by activating transcription of the early genes (encode MS ring, C ring, and export apparatus, FlbD, FliX). Additionally, CtrA controls expression of *rpoD* [79] and is needed for transcription of the early flagellar genes [80]. FlbD and FliX are transcriptional regulators that regulate expression of the early and middle genes. The middle and late genes, which encode the hook protein and flagellins, are transcribed only after the components encoded by the early genes have been assembled into the nascent flagellum. FlbD is an RpoN-dependent transcriptional activator which binds *ftr* (flagella transcriptional regulation) sequence motifs to regulate gene expression. Once the early genes are expressed, FlbD binds the *ftr* sequence motifs in the promoter regions of early flagellar genes to inhibit transcription of these genes. For example, FlbD binds the *ftr*4 site located upstream of the early gene *fliF *to repress its transcription once the early genes have been expressed [81]. In contrast, FlbD stimulates transcription of the middle genes by binding to the *ftr* sites of the middle and late genes [81, 82]. FlbD activity is regulated by FliX, which acts to either inhibit or stimulate FlbD. In the absence of early flagellar structures, FliX inhibits FlbD activity by binding FlbD preventing it from binding *ftr* sites [83]. Once the early flagellar structures are formed FliX stimulates FlbD. The mechanism for this stimulation is unknown, but it may involve another factor such as an assembled component of the flagellum which converts FliX into a positive regulator, or it may involve covalent modifications of FliX or FlbD [83]. Although FlbD possesses an N-terminal response regulator domain, FlbD activity does not appear to be regulated by phosphorylation via a sensor kinase. The exact cellular cues that regulate FlbD are not known, but FlbD activity is linked to cell division [84].

Unlike the bacteria discussed thus far, flagellar biogenesis in *C*. *crescentus* does not involve FliA. Rather, the six flagellin genes found in *C*. *crescentus* are RpoN dependent. The expression of the flagellin genes is regulated by FlbD and FlbT which binds the 5′ UTR of flagellin transcripts and represses translation until the hook-basal body structure is assembled [85]. Though the mechanism for this temporal regulation is unknown, one possibility is that FlbT represses translation of flagellin genes until a positive factor binds the 5′ UTR region of flagellins to promote translation upon assembly of the mature hook-basal body structure [85].

## 3. Coupling Flagellar Biosynthesis with Other Cellular Activities

Though flagellar biogenesis is a complex process in itself, it is further complicated by its coordination with other cellular processes. Flagellar biosynthesis is timed with other cellular activities such as cell replication and quorum sensing. Environmental stimuli also play a role in regulating flagellar biogenesis as bacteria respond to changes in the environment by altering behavior needed for survival. Many of these responses involve altering the state of flagellar biogenesis within these different contexts.

### 3.1. Modulating Flagellar Gene Expression during the Cell Cycle

Flagellar biosynthesis is linked to the cell cycle through various mechanisms and involves coordinating flagellar gene expression with growth phase and cell division. In *V. cholerae*, chemotaxis and motility genes are upregulated as the population enters stationary phase. Of the 114 genes identified with roles in chemotaxis and flagellar biogenesis, 72 genes were upregulated more than 2-fold during stationary phase compared to mid-exponential phase [86]. The upregulation of 60 of the 72 chemotaxis and flagellar genes is dependent on RpoS, indicating a correlation between flagellar biogenesis and entry into stationary phase [86]. The coordination between entry into stationary phase and flagellar biogenesis may aid the bacterium in exiting the host when nutrients become depleted and prepare the bacterium for colonization of other niches or survival in environmental reservoirs.

*C*.* crescentus* is a particularly good organism for studying how flagellar biogenesis is integrated with the cell cycle because of its two distinct cell morphologies. The flagellar filament is assembled just prior to cell division and all progeny swarmer cells possess a fully functional flagellum. The synthesis of the flagellum is intimately linked to the cell cycle through regulatory proteins such as CtrA, FlbD, and FliX that are involved in both the cell cycle and flagellar biogenesis. This dual functionality may exist to ensure that each swarmer cell is flagellated. The master regulator for flagellar biogenesis, CtrA, regulates at least 95 genes [87], including the cell-division genes *ftsZ* [88] and *ftsQA* [89]. Cells of a *fliX* mutant become filamentous as they enter late log phase [90] demonstrating a role for this flagellar regulatory protein in cell division. Flagellar biogenesis is also linked to the cell cycle via FlbT and FlaF. FlaF is required for flagellar biogenesis and motility [91]. FlbT protein levels are constant throughout the cell cycle, while FlaF levels correlate to flagellin levels, both of which peak just prior to cell division [91]. Since FlbT is a negative regulator for flagellin translation and peaks in flagellin levels correlate with FlaF levels, this observation suggests that FlaF may temporally modulate FlbT activity [91].

Expression of *Vibrio vulnificus* FlhF, which is needed for polar localization of the flagellum, is regulated by the quorum sensing master regulator SmcR. *flhF* transcript levels in a *smcR* mutant are higher than wild-type levels indicating that SmcR represses *flhF* expression [92]. In wild-type cells, *flhF* transcript levels are highest during exponential phase and decrease upon entry into stationary phase [92]. In contrast, transcript levels of *smcR* increase as cultures enter stationary phase suggesting that SmcR plays a major role for growth phase-dependent variation of *flhF* expression [92]. This growth phase-dependent gene expression may be important in host colonization as during the initial infection bacterial cell densities are low but at later stages of colonization when bacterial cell densities are higher motility may not be necessary.

Expression of flagellar genes is regulated in *P. fluorescens* throughout the growth phases by the Gac (GacA/GasS) two-component system which limits flagellar biosynthesis during exponential growth by downregulating transcription of *fleQ *(encodes the master regulator) [93]. The Gac system positively regulates production of virulence factors and quorum sensing molecules and is required for full virulence in animal and plant hosts [94]. Levels of the Gac system components are regulated in response to growth phase as *gacA* and *gacS* transcript levels are highest at mid-exponential growth phase [95]. The concomitant upregulation of virulence factors and the downregulation of flagellar genes by the Gac system may be a way to coordinate virulence and motility with growth phase so that virulence factors are expressed at high cell densities during host colonization and flagellar genes are turned off to facilitate attachment to the host.

### 3.2. Connections between Quorum Sensing, Host Colonization, and Flagellar Gene Expression

In many pathogenic bacteria, flagella aid in surface attachment and colonization. In such cases, successful colonization may require cooperation between quorum sensing and flagellar synthesis to integrate surface attachment and virulence gene expression at high cell densities in pathogens. Surface colonization can lead to biofilm formation which is generally more resistant to host defenses and antimicrobials. Pathogens use different mechanisms to couple expression of virulence genes with flagellar gene expression in the context of the host. This includes modulating quorum sensing and host colonization based on the status of the flagellum or modulating flagellar biogenesis based on quorum sensing signals.

Quorum sensing is used to regulate gene expression in response to cell density. This is achieved through the secretion of a signaling molecule called an autoinducer (AI). AIs are produced by bacteria and travel across the cell membrane and accumulate in the environment. Upon reaching a critical concentration, the AIs stimulate transcription of genes required for group behavior. *luxS* is responsible for the production of AI-2 [96] and an *H*. *pylori* mutant in *luxS* exhibits decreased motility compared to wild type [97]. *flhA* transcript levels are decreased in the *luxS* mutant, but addition of 4, 5-dihydroxy-2,3-pentanedione (functions as AI-2) into the growth medium restores *flhA* transcript levels to wild-type levels [97]. Like the *H. pylori luxS* mutant, a *C*. *jejuni luxS* mutant is less motile than wild type [98] and the mutant displays reduced transcript levels of *flaA* [99]. Using DNA microarrays, He and coworkers found that a majority of the flagellar genes are downregulated in a *C*. *jejuni luxS* mutant [98]. Expression of chemotaxis genes, however, is unchanged in the *luxS* mutant indicating that the motility defect of the *luxS* mutant is due to defects in flagellar biogenesis and not chemotaxis [98]. A rationale for the link between cell density and flagellar biogenesis is that it may aid in colonization of host tissue by ensuring that a sufficient number of flagellated bacteria are present to establish a successful infection.

Regulatory proteins involved in flagellar biogenesis can regulate quorum sensing and colonization or they themselves can be regulated by quorum sensing and colonization factors. For example, the RpoN-dependent activator FlrC not only is required for flagellar biogenesis but also appears to play a role in host colonization by *V*. *cholerae*. Mutants expressing a FlrC variant incapable of being phosphorylated fail to produce flagella and are therefore defective in colonization [54]. Interestingly, a mutant expressing a constitutively active form of FlrC is also deficient in colonization even though the cells are motile and produce flagella [54]. This observation demonstrates the need for coordinating flagellar biogenesis and pathogenesis for successful colonization. Conversely, Vfr, a major quorum sensing and virulence regulator in *P*. *aeruginosa*, downregulates expression of flagellar genes by repressing transcription from the *fleQ* promoter [100]. Inhibition of flagellar biogenesis by Vfr may aid *P. aeruginosa *in persisting at the site of infection to achieve high cell densities and virulence gene expression.

HapR is a transcriptional regulator involved in quorum sensing in *V*. *cholerae* which also regulates expression of virulence genes. HapR represses expression of several virulence genes [101] and *hapR* expression is regulated by an unknown mechanism involving FliA [102]. *hapR* expression is depressed in *flgM* and *flgD* mutants (both of these mutations alleviate FlgM repression on FliA activity), while deletion of *fliA* results in elevated *hapR* expression [102]. The authors of this study speculated that expression of HapR is regulated by a regulator or small, noncoding RNA (sRNA) that is part of the FliA regulon. FliA-mediated regulation of *hapR* appears to be linked to the shearing of flagella from bacteria that penetrate the mucin layer to infect the host. This shearing of the flagella leads to secretion of FlgM via the flagellar protein export apparatus and results in increased expression of FliA-dependent genes and repression of *hapR* expression [102]. The outcome of such a mechanism is that virulence genes which are negatively regulated by HapR are expressed specifically when *V. cholerae* penetrates the intestinal mucous layer. *hapR* production is also regulated by the quorum sensing protein LuxO, further linking virulence to cell density. LuxO is an RpoN-dependent activator [103] which, along with HapR, regulates motility, protease production, and biofilm formation [101]. High cell densities are common during late stages of infection and the HapR-mediated repression of virulence genes may aid *V. cholerae* in detaching and finding new colonization sites [86, 101].

*V*. *parahaemolyticus* uses its polar flagellum to detect surfaces by sensing impedance to the polar flagellum rotation rate [104]. Once a surface is encountered, expression of the lateral flagellar genes is induced. Lateral flagella are important for host colonization as mutants that do not produce lateral flagella are deficient in adherence to HeLa cells and biofilm formation [61]. Gode-Potratz and coworkers identified ~70 genes that are surface responsive, most of which are positively regulated [64]. Some of the surface-regulated genes that are upregulated are virulence genes, such as a putative GbpA homolog and components of a type III secretion system [64]. GbpA from *V*. *cholerae* promotes adherence to chitinous surfaces of zooplankton and human epithelia cells [105] which is consistent with the observed upregulation of *gbpA* in *V*. *parahaemolyticus* when it encounters a surface.

The *P*. *aeruginosa* flagellum is an important determinant in the susceptibility of the bacterium to host defenses. *P. aeruginosa fliC* mutants are unable to upregulate transcription of *lasI* and *rhlI* which encode enzymes that synthesize quorum sensing homoserine lactones [106]. These quorum sensing molecules regulate production of exoproteases, which degrade surfactant protein-A (SP-A), an antimicrobial that opsonizes and permeabilizes membranes of lung pathogens [106]. In the absence of these exoproteases, the *P*. *aeruginosa fliC* mutants are cleared from the lung [106]. Though it is not known why *fliC* mutants are unable to upregulate transcription of *lasI* and *rhlI*, it is not due to the inability to sense the environment [106]. *P. aeruginosa fliE*,* flgE*, *fliC*, and *fliD* mutants are also attenuated in LPS biosynthesis which compromises the integrity of their outer membranes making them more susceptible to SP-A [107]. Additionally, *flgE* and *fliC* mutants make less pyocyanin, a redox-active toxic secondary metabolite that is crucial for lung infection [108, 109].

### 3.3. Reacting to pH Changes by Gastrointestinal Pathogens

Gastrointestinal pathogens encounter varying environmental conditions as they travel from the mouth to the stomach and then to the intestines. The gastric pathogen *H*. *pylori* has the added burden of long-term survival in the acidic environment of the stomach. Pathogens that colonize the intestinal tract must also survive passage through the stomach in high enough numbers to be able to colonize their target organs. Thus, it is not surprising that gastrointestinal pathogens often modulate gene expression in response to acidic conditions.

Merrell and coworkers found that expression of approximately 7% of the *H*. *pylori* genes is altered by a shift to low pH [110]. Many of these genes are involved in flagellar synthesis with the majority of those being RpoN dependent [110]. FliA-dependent genes, such as *flgM* in *H*. *pylori* [110] and *flaA* in *C*. *jejuni *[111], are also upregulated at low pH conditions. The upregulation of flagellar genes is critical for survival of *H. pylori* as the bacterium is not able to survive at low pH but must swim across the mucous layer to the underlying gastric epithelium to establish a successful infection. In addition to enhancing transcription of flagellar genes, acidic environments also stimulate motility and swimming speeds, potentially in response to increased concentration of protons in the stomach [110].

The basis for upregulation of the RpoN-dependent flagellar genes in *H. pylori* likely involves a stimulation of FlgS activity at low pH. Wen and coworkers reported that *H. pylori *requires FlgS to survive acid stress (30-minute exposure to pH 2.5) [112]. These researchers found that, besides the known RpoN-dependent flagellar genes, FlgS was required for the upregulation of 86 genes in response to acid stress, many of which were previously known to be required for acid stress survival [112]. It is not known if FlgS responds directly to acid stress or if the stress signal is mediated through another factor.

### 3.4. Altering Flagellar Biogenesis by Phase Variation

In some bacteria flagellar biogenesis is subject to phase variation events that affect expression of specific flagellar genes. Phase variation causes changes in phenotypes at frequencies that are much higher than random mutations and contribute to heterogeneity within a population. The ability of *H*. *pylori* to persist in the host may involve the phase-variable dependent expression of flagella that can serve as an advantage for adaptation to the host and for evading the host immune response. *C*. *jejuni* utilizes phase variation to turn on and off production of flagella, a strategy that increases commensal colonization in poultry [113]. Many phase variation events occur by random reversible changes in the length of short DNA sequence repeats resulting from slipped-strand mispairing. These events can take place within the coding sequence or in the promoter of a gene. *C. jejuni* phase variants in which expression of *flgR* is in the OFF state result from the addition or removal of a nucleotide within one of two homopolymeric nucleotide tracts consisting of adenine and thymine. These nucleotide tracts are located within the coding sequence of *flgR* causing the sequence to shift out of frame [113]. Revertants to the ON state arise upon the addition or removal of a nucleotide within the original mutated homopolymeric nucleotide tract to restore the wild-type sequence. Alternatively, pseudorevertants can arise in which nucleotides are removed or added close to the original mutated homopolymeric nucleotide tract to restore the correct reading frame but which result in changes in the amino acid sequence of the protein [113]. *flgS* is also regulated by phase variation in *C*. *jejuni*, providing multiple levels of phase-variable control for the RpoN regulon [114, 115]. Similar mechanisms of phase variation also exist for *flhA* in *C*. *coli *[116] and *fliP* in *H*. *pylori* [117] which leads to the formation of truncated proteins that prevent flagellar biogenesis.

A second type of phase variation in flagellar biogenesis is mediated through alterations in DNA methylation which impact the expression of specific flagellar genes. Host-adapted bacterial pathogens, such as *H*. *pylori*, often possess DNA methyltransferases and type III restriction-modification (R-M) systems that contain simple tandem DNA repeats which undergo slipped-strand mispairing during DNA replication that causes frameshift mutations and phase variation [118]. R-M systems, widespread among many bacteria, confer protection from invasion by foreign DNA. Type III R-M systems are composed of a methyltransferase (*mod*) and an endonuclease (*res*) gene, whose gene products typically function together. The switching between ON and OFF states of a DNA methyltransferase can regulate expression of a “phasevarion,” via differential methylation of the genome in ON and OFF states [119]. Many of these phase-variable methyltransferase genes are associated with an inactive *res* gene resulting from frameshift mutation or are orphans that are not associated with a *res* gene, suggesting that DNA restriction is not the primary role of these methyltransferases (reviewed by Fox et al. [120]). For example, *H. pylori modH* encodes a phase-variable DNA methyltransferase and the cognate *res* gene contains a nonsense mutation, frameshift mutation or is absent in various *H. pylori* strains [119]. *modH* OFF strains have decreased expression of *flaA* and *fliK* compared to the *modH* ON strain [119]. FlaA has a low ability to activate innate immunity via the Toll-like receptor 5 [121] and modulating expression of the flagellin may be advantageous in evading the host response. Another *H*. *pylori* methyltransferase with an inactive *res* gene is *hpyAVIBM *[122, 123]. *hpyAVIBM *encodes a C^5^ cytosine methyltransferase and contains AG repeats in its open reading frame, making it susceptible to phase variation via frame shift mutations [124]. For instance, *hpyAVIBM* from strain 26695 and *hpyAVIBM* from strain HPAG1 have five AG repeats whereas the four AG repeats in strain San 74 result in the translation of a truncated protein [124]. Deletion of *hpyAVIBM* results in both the upregulation and downregulation of *rpoN*, *fliR*, *fliD*, *fliS*, *motA*, *fliK*, and *flgK *depending on the strain used [124] indicating that phase variation of *hpyAVIBM* can alter flagellar biogenesis.

*P*. *aeruginosa* populations can possess two phenotypic variants, one which forms small, rough colonies (S) and another which forms large, flat (L) colonies. S variants form biofilms in nonagitated liquid cultures whereas the L variants do not [125]. S variants possess defects in flagellum-mediated swimming, flagellum-mediated swarming, and type IV-pilus-mediated twitching but are able to revert back to the L phenotype at relatively high frequencies suggesting that the shift between phenotypes is regulated by phase variation [125]. Phase variation also occurs in *P. fluorescens* which colonizes the alfalfa rhizosphere. Phenotypic variants arise during rhizosphere colonization and are dependent on the activity of a site-specific recombinase [126]. C variants have wild-type colony morphology while F and S variants have a translucent and diffuse colony morphology [126]. F and S variants, which colonize distal parts of the roots and swim faster than the C variants, overproduce *fliC* (flagellin) transcripts and synthesize flagellin filaments that are ~3 times longer than those of the C variant [126].

## 4. Conclusions and Future Directions

Flagellar biogenesis is a carefully choreographed process in which many different components of the flagellum are made as they are needed for assembly into the nascent flagellum. Bacteria have evolved elaborate and sundry regulatory networks to couple flagellar gene expression with assembly. A driving force for the evolution of such regulatory networks is undoubtedly the environmental and ecological constraints imposed by a particular bacterium's niche. In addition, the degree to which a given bacterial species needs to couple flagellar biogenesis to other cellular processes, such as cell division, has also likely shaped the evolution of these regulatory networks. We focused here on bacteria which utilize RpoN for flagellar biogenesis; yet even among these bacteria the mechanisms used to regulate expression of the RpoN-dependent flagellar genes vary greatly. For most of the systems described in this review we have only scratched the surface in our understanding of how they operate. For each of these systems, fundamental questions remain to be answered such as the following: are all of the flagellar genes of a given system expressed as part of a transcription hierarchy or are some genes expressed constitutively? Are there regulatory mechanisms within RpoN flagellar regulons that fine-tune gene expression so that components of the flagellum are made precisely when needed? What are the cellular cues sensed by the regulatory proteins that control expression of the RpoN-dependent flagellar genes? How does the flagellar export apparatus control expression of RpoN-dependent flagellar genes in various bacteria? Does FlgZ have a regulatory role in flagellar biogenesis in *H. pylori* and other bacteria? As our understanding of the molecular genetics of diverse bacterial species expands, novel regulatory mechanisms that control expression of flagellar genes within these bacteria will undoubtedly come to light. For example, it was only recently that sRNAs were identified in *H. pylori*, some of which are potentially FliA dependent [127] and may have roles in flagellar biogenesis. Finally, with increasingly sophisticated methods in bioinformatics, genomics, and proteomics, further characterization of flagellar regulatory networks will provide important insights into the mechanisms governing gene expression and identify new regulatory proteins and mechanisms.
